# Anterior segment oculomics in systemic sclerosis: reduced OCT-derived pupil diameter without iris morphology alterations

**DOI:** 10.3389/fmed.2026.1776963

**Published:** 2026-08-07

**Authors:** Aleksandra Leoniuk, Anna Szwedowicz, Barbara Pieklarz, Ewa Gińdzieńska-Sieśkiewicz, Otylia Kowal-Bielecka, Joanna Konopińska, Diana A. Dmuchowska

**Affiliations:** 1 Ophthalmology Department, Medical University of Bialystok, Bialystok, Poland; 2 Department of Rheumatology and Internal Diseases, Medical University of Bialystok, Bialystok, Poland

**Keywords:** anterior segment optical coherence tomography, autonomic nervous system, iris cross-sectional area, iris thickness, microvascular dysfunction, oculomics, pupil diameter, systemic sclerosis

## Abstract

**Background:**

Systemic sclerosis (SSc) is a multisystem autoimmune disease characterized by microvascular dysfunction and fibrosis. While posterior segment ocular involvement has been described, quantitative anterior segment alterations remain poorly characterized. The iris, as part of the uveal tract, shares vascular features with the choroid and may reflect systemic microvascular and autonomic dysregulation. Within the emerging framework of oculomics, anterior segment optical coherence tomography (AS-OCT)–derived iris and pupil metrics may represent accessible, non-invasive ocular features of systemic neurovascular health.

**Objectives:**

To compare static AS-OCT-derived pupil diameter, iris thickness, and iris cross-sectional area between patients with SSc and healthy controls, and to explore their associations with systemic and ocular parameters.

**Methods:**

In this prospective cross-sectional study, 31 patients with SSc and 32 age- and sex-matched healthy controls underwent standardized AS-OCT imaging under controlled photopic conditions. Pupil diameter, iris thickness at 1, 2, and 3 mm from the pupil margin, and nasal and temporal iris cross-sectional areas up to 3 mm from the pupil margin were quantified using ImageJ software. Inter-rater reproducibility was assessed using intraclass correlation coefficients. Group comparisons and exploratory correlation and univariate regression analyses were performed.

**Results:**

Iris thickness and cross-sectional areas did not differ significantly between groups. Static AS-OCT-derived pupil diameter was smaller in the SSc group in the unadjusted comparison (*p* = 0.033); however, after adjustment for mean arterial pressure (MAP) this difference was no longer statistically significant, although the direction and magnitude of the effect were preserved (*β* = −366.9 μm, *p* = 0.057). In controls, pupil diameter correlated positively with selected iris parameters, whereas such associations were absent in SSc. Exploratory correlation and regression analyses did not provide consistent evidence of structural anterior uveal involvement.

**Conclusion:**

Under standardized acquisition conditions, static AS-OCT pupil diameter tended to be smaller in patients with SSc, but this difference did not remain statistically significant after adjustment for MAP. Structural iris parameters did not differ between groups. This preliminary, hypothesis-generating finding may be consistent with functional rather than structural anterior segment alterations, but it does not directly demonstrate autonomic or neurovascular dysfunction and requires validation in larger cohorts, preferably with dynamic pupillometry.

## 1 Introduction

Systemic sclerosis (SSc) is a chronic, multisystemic autoimmune connective tissue disease associated with progressive fibrosis and widespread vasculopathy. Although the etiology of the disease remains unknown, its pathogenesis is multifactorial. It involves early microvascular injury, immune system activation, chronic inflammation, and excessive extracellular matrix deposition, culminating in systemic fibrosis. These mechanisms lead to persistent tissue hypoxia and ischemia, which affects the skin and a variety of internal organs including the lungs, heart, gastrointestinal tract, kidneys, and musculoskeletal system (1–5). SSc is clinically divided into two primary subtypes based on skin involvement: diffuse cutaneous SSc (dcSSc) and limited cutaneous SSc (lcSSc). In dcSSc, skin fibrosis extends beyond the distal limbs to involve the upper arms, thighs, and trunk. In contrast, lcSSc concerns the skin of the distal extremities and often includes the face and neck (4). DcSSc typically has a worse prognosis due to more severe internal organ involvement. Importantly, ocular manifestations may be present in both subtypes, because of the systemic nature of microvascular dysfunction and fibrosis in SSc (4, 6).

From an ophthalmological standpoint, SSc can affect multiple ocular structures, such as the eyelids, lacrimal glands, conjunctiva, cornea, anterior chamber, retina, choroid, optic nerve, and extraocular muscles. Patients may present keratoconjunctivitis sicca, eyelid tightening, or restricted eye movements due to periorbital skin fibrosis (2, 3, 7). Moreover, microvascular dysregulation related to SSc may contribute to retinal and choroidal vascular alterations, such as changes in choroidal thickness and choroidal hypoperfusion. It was demonstrated by optical coherence tomography (OCT) and OCT angiography (OCTA) in many studies (8–19).

In our previous studies on SSc we focused our analysis on the posterior segment of the eye. We demonstrated reduced macular and peripapillary choroidal thickness and altered choroidal vascularity indices in patients with SSc, indicating choroidal involvement as an indicator of microvascular damage (16, 17). We also showed high interocular symmetry of choroidal thickness and CVI in SSc, supporting the reliability of unilateral measurements in both research and clinical practice (18). We found that despite significant choroidal alterations, retinal layer thickness remained largely preserved with only subtle associations between choroidal and outer retinal parameters, suggesting that choroidal compromise may precede overt structural retinal changes (19).

In recent years, there has been growing interest in oculomics, which explores ocular imaging as a non-invasive window into systemic microvascular health across a range of diseases (20). Within this framework, OCT-derived parameters from the posterior segment, particularly choroidal metrics, have been increasingly investigated as potential ocular markers of systemic small-vessel involvement. Anterior segment optical coherence tomography (AS-OCT) is the primary noninvasive tool for visualizing the iris morphology. Accordingly, our group has focused on evaluating OCT-based biomarkers from the posterior segment of the eye as indicators of systemic microvascular pathology. In addition to SSc, we examined choroidal alterations in diabetic retinopathy and pulmonary hypertension, demonstrating significant disease-related changes in choroidal parameters (16, 18, 19, 21, 22).

Given posterior segment involvement in SSc and other small-vessel diseases, it is reasonable to examine whether similar alterations extend to the anterior uveal tract. As the iris represents the anterior component of the uveal system and shares vascular characteristics with the choroid, it may likewise reflect systemic microvascular pathology. The iris is composed of several layers, which from anterior to posterior include: the anterior border layer, the stroma, the dilator muscle (anterior myoepithelium), and the iris pigment epithelium (23, 24). Reported prevalence of iris alterations in SSc varies with iris pigmentation and the assessment method used. Iris abnormalities described in SSc include transillumination defects and vascular irregularities, which are more readily detected in individuals with lighter pigmented irides (gray or blue) (25). Overall, iris alterations have been reported in 8.9–13.7% of SSc patients (26, 27). These findings are thought to reflect vasculopathy affecting the iris as part of the uveal tract, potentially resulting in microvascular dysfunction with stromal and pigment epithelial involvement (27, 28).

Pupil diameter is primarily governed by autonomic balance between sympathetic (mydriasis) and parasympathetic (miosis) activity. Autonomic involvement has been reported in SSc, and pupillometry studies have suggested altered resting pupil size and reactivity in this disease. These observations support pupil diameter as a pragmatic anterior segment metric potentially reflecting systemic neurovascular dysregulation (29–33). Importantly, pupil diameter can be obtained directly from standard AS-OCT scans acquired in many clinical settings, without the need for dedicated pupillometry equipment. This makes OCT-based pupil diameter a pragmatic and broadly accessible metric that can be integrated with simultaneous iris morphometry.

Despite these observations, quantitative OCT-based analysis of pupil diameter and iris morphology has not been yet performed in SSc. Because the iris shares key vascular features with the choroid, OCT-derived metrics such as pupil diameter, iris thickness, and cross-sectional area may serve as ocular markers of microvascular involvement and autonomic dysregulation.

This study aimed to quantify pupil diameter, iris thickness, and iris cross-sectional area in patients with SSc compared with healthy controls, to explore potential anterior segment ocular markers related to systemic disease features. The prespecified primary endpoint was OCT-derived pupil diameter measured under standardized acquisition conditions, selected as a pragmatic static parameter consistent with potential autonomic involvement in SSc. Secondary and exploratory endpoints included iris thickness (at 1, 2, and 3 mm from the pupil margin) and nasal/temporal iris cross-sectional area (up to 3 mm from the pupil margin). We hypothesized that individuals with SSc would exhibit alterations in pupil diameter and anterior iris parameters relative to controls, consistent with chronic microvascular involvement and possible autonomic dysregulation.

## 2 Materials and methods

### 2.1 Study design and participants

This cross-sectional, prospective study was carried out between 2021 and 2024 at the Department of Ophthalmology and the Department of Rheumatology and Internal Medicine, Medical University of Bialystok.

A total of 31 adult patients diagnosed with SSc and 32 healthy controls were enrolled. The study protocol was approved by the Bioethics Committee of the Medical University of Bialystok (APK.002.109.2021) and was conducted in accordance with the Declaration of Helsinki. All participants provided written informed consent prior to inclusion.

### 2.2 Ophthalmological examination

All patients underwent a comprehensive ophthalmological examination. The examination included refraction, best corrected visual acuity (BCVA) assessed using a Snellen chart and converted to logMAR, and slit-lamp biomicroscopy. Axial length and pachymetry were measured using a Tomey OA-2000 biometer (Nagoya, Japan). Intraocular pressure (IOP) was assessed with a Pascal dynamic contour tonometer (DCT; Zeimer Ophthalmic Systems AG, Port, Switzerland), and fundus evaluation was performed following pharmacological dilation. Optical coherence tomography (OCT; Heidelberg Engineering GmbH, Heidelberg, Germany, 2016) was used to assess the retina, choroid and anterior segment of the eye. Blood pressure was measured after 5 min of seated rest.

### 2.3 Clinical data

Diagnosis was established in accordance with the 2013 ACR/EULAR classification criteria for SSc with patients subsequently categorized into diffuse or limited subtypes (34–36). Collected data included demographic information (age, sex), SSc subtype (limited or diffuse), disease duration, autoantibody profile (anti-Scl-70, anti-centromere), smoking status, and systemic treatment. Clinical features recorded were history of digital ulcers, joint involvement (arthralgia/swelling), interstitial lung disease confirmed by HRCT, and cardiac involvement indicated by elevated NT-proBNP or fibrosis on MRI. Inflammatory markers (CRP, ESR) were measured. Structural microvascular changes were assessed noninvasively by nailfold capillaroscopy (NFC) using a CapillaryScope 200 Dino-Lite microscope. NFC patterns were classified as early, active, or late SSc according to capillary morphology, density, size, and hemorrhage presence, following established criteria. Participants in both SSc and control groups were divided based on iris pigmentation (green/blue and brown/brown-green) to minimize the effect related to melanin differences in OCT image reflectivity on measurement accuracy. Sidhartha et al. found that darker iris color was associated with greater iris thickness at 750 μm from the scleral spur (37).

### 2.4 Inclusion and exclusion criteria

Inclusion criteria included age above 18 years and consent to participate in the study. Patients in the SSc group met the 2013 ACR/EULAR criteria for SSc, and the control group comprised self-reported healthy volunteers (34, 35).

Exclusion criteria: ametropia ≥3 diopters, presence of any fundus pathology or macular abnormalities, history of posterior segment surgery or intravitreal injections, retinal laser treatment, diabetes, phacoemulsification performed within 12 months prior to examination, and poor quality of the OCT scans (<30 dB), use of medications known to affect pupil diameter (systemic or topical), including anticholinergics, sympathomimetics, opioid analgesics, and topical mydriatics/miotics.

### 2.5 OCT acquisition, image processing, and measurements

Anterior segment imaging was performed using a spectral-domain OCT device (Spectralis OCT; Heidelberg Engineering, Heidelberg, Germany, 2016) with the anterior segment module in angle mode. Scans were acquired under standardized photopic illuminance conditions (230 lux) with no pharmacological mydriasis. We did not administer dilating drops because they are known to reduce the radial length of the iris and alter its thickness during mydriasis (37). Bright light conditions were applied to induce miosis, thereby reducing image irregularities and minimizing motion artifacts related to iris sphincter activity. Illuminance was measured using a digital light meter (UT383S, UNI-T, Uni-Trend Technology, Dongguan, China). After a 5-min light adaptation period (230 lux), pupil diameter was assessed (μm). During measurement sessions, each subject was requested to focus on a standard fixation point. All scans were acquired between 12 p.m. and 3 p.m. to minimize the influence of diurnal variations on measurements by the same experienced examiner (BP). Imaging was performed prior to pharmacological dilation required for fundus examination (no mydriatic drops were instilled before AS-OCT).

**Figure 1:**
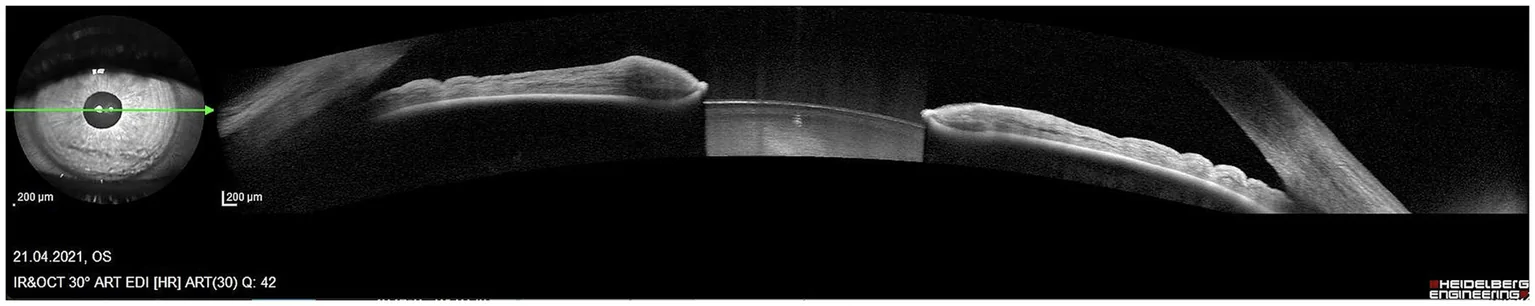
Single horizontal OCT B-scan (30°) centered on the pupil.

A single horizontal B-scan (30°) centered on the pupil was obtained for each eye. Scans were accepted for analysis only if the nasal and temporal iris and the anterior chamber angle were clearly visible, without motion artifacts, and with signal quality >30 dB (Figure 1).

The Spectralis AS-OCT module does not provide an automated segmentation algorithm for the evaluation of iris thickness and cross-sectional area. The only measurement tool available is a manual point linear caliper. While this approach is appropriate for tissues with smooth and regular boundaries, it is less reliable for the iris, which presents irregularities such as crypts and surface undulations, thereby limiting the accuracy of vertical measurements. To overcome this limitation, OCT image evaluation was performed using ImageJ software^1^ (Supplementary material, Step-by-Step Measurement Description). Minor variations in scan orientation are inherent to clinical AS-OCT acquisition. Its potential impact was minimized by dual-axis calibration, local perpendicular measurements, and area-based segmentation.

**Figure 2:**
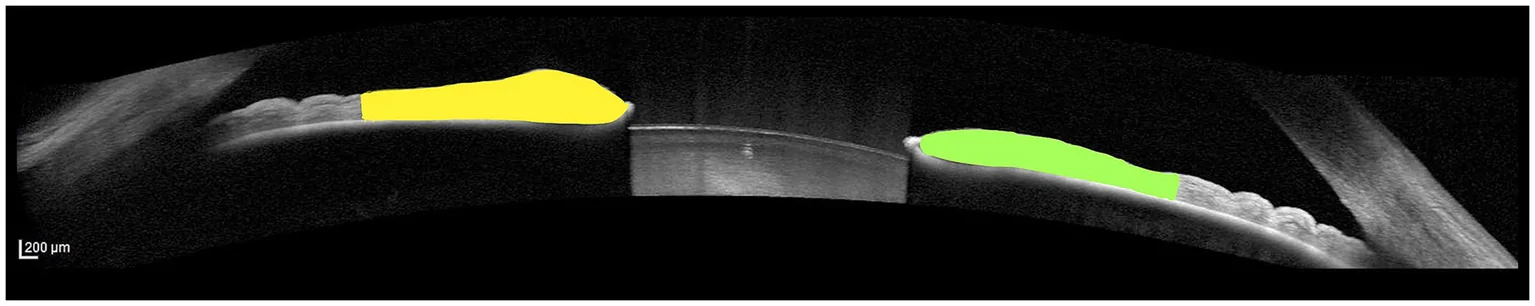
Schematic illustration of nasal and temporal iris cross-sectional area measurements. The nasal iris area is marked in yellow and the temporal iris area in green. Iris cross-sectional area was measured from the pupillary margin to a line perpendicular to the local iris surface, located 3,000 μm from the pupillary margin, separately in the nasal and temporal sectors. The area was bounded anteriorly by the anterior iris surface and posteriorly by the anterior border of the iris pigment epithelium.

**Figure 3:**
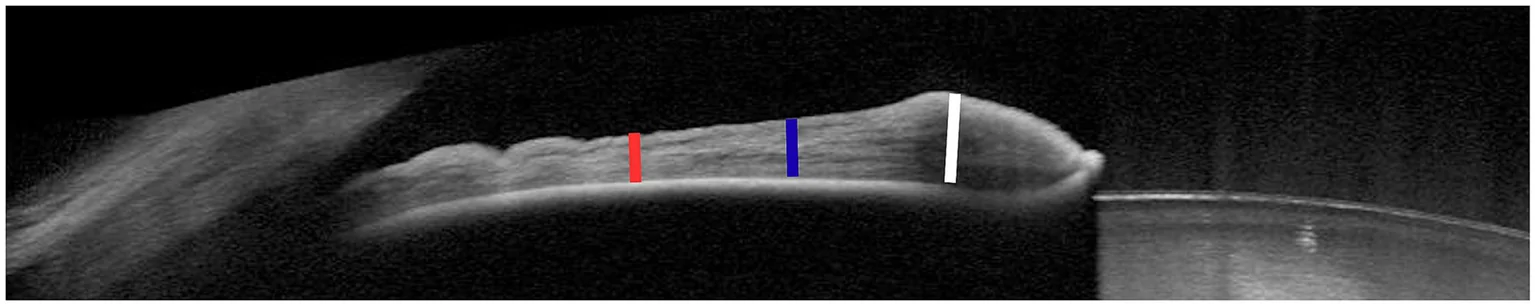
Schematic representation of nasal iris thickness measurements at 1,000 μm, 2,000 μm, and 3,000 μm from the pupillary margin. The corresponding measurement lines are shown in white, blue, and red, respectively. At each location, a line perpendicular to the local iris surface was drawn, and iris thickness was measured from the anterior iris surface to the anterior border of the iris pigment epithelium.

The cross-sectional area of the iris was manually outlined along reference lines located 3,000 μm from the pupil margin, either nasally or temporally. ImageJ quantified the number of pixels within each manually defined region of interest, which, after applying the micrometer-per-pixel calibration, yielded the corresponding area (Figure 2). Additionally, iris thickness was measured at distances of 1,000 μm, 2,000 μm, and 3,000 μm from the pupil margin, and pupil diameter was assessed (Figure 3). Iris thickness was measured from the anterior iris surface to the anterior border of the iris pigment epithelium (IPE). Due to limited OCT penetration and reduced delineation of posterior borders in highly pigmented tissues, the posterior iris pigment epithelium was not included in thickness and cross-sectional area measurements (24).

### 2.6 Reproducibility

All measurements were performed independently by two evaluators blinded to clinical data (AL, AS). Inter-rater agreement for all iris parameters was assessed using the intraclass correlation coefficient (ICC) with 95% confidence intervals. Given the excellent agreement between the evaluators (Table 1), the average of the two measurements was used for subsequent analyses.

**Table 1:** Inter-rater reproducibility of AS-OCT-derived pupil and iris measurements.

Parameter		ICC (95% CI)
Pupil diameter [μm]		0.999 (0.999–1.000)
Iris thickness [μm]	1 mm T	0.973 (0.935–0.986)
	2 mm T	0.983 (0.972–0.990)
	3 mm T	0.992 (0.986–0.995)
	1 mm N	0.976 (0.906–0.990)
	2 mm N	0.985 (0.963–0.993)
	3 mm N	0.986 (0.976–0.991)
Iris cross-sectional area [μm^2^]	T	0.990 (0.975–0.995)
	N	0.988 (0.976–0.993)

*iris thickness measured without iris pigment epithelium. *iris cross-sectional area measured up to 3 mm from pupil margin. ICC, Intraclass correlation coefficient; N, nasal; T,temporal; 1/2/3 mm, distance from pupil margin.

### 2.7 Choroidal parameters

Choroidal parameters used in the present study were retrieved from the dataset previously generated for our published analyses of macular and peripapillary choroidal parameters in patients with SSc and healthy controls, with methodology described in detail elsewhere. These EDI-OCT metrics included macular choroidal thickness, volume and CVI as well as peripapillary choroidal thickness and CVI. The central macular region was defined as the central ETDRS subfield, corresponding to a circular area with a radius of 500 μm from the foveal center (16, 17).

### 2.8 Statistical analysis

For each participant, one eye per participant was randomly selected for evaluation. The study eye was selected using simple computer-generated randomization with equal probability assigned to the right and left eye. The randomization was performed before image analysis and independently of group allocation. Statistical analysis was conducted using R 4.2.1. statistical software [R Core Team (2022). R: Language and environment for statistical computing by R Foundation for Statistical Computing, Vienna, Austria]. All calculations were based on two-sided *α* = 0.05. All statistical tests were two-sided. The prespecified primary endpoint was OCT-derived pupil diameter. Iris thickness, iris cross-sectional area, correlation analyses, and univariate regression analyses were considered secondary or exploratory. No formal adjustment for multiple comparisons was applied to exploratory analyses; therefore, these results were interpreted as hypothesis-generating.

Analysis of agreement between both evaluators was based on ICC (Intraclass correlation coefficient), with 95% confidence interval (CI). Two-way model, absolute agreement and single rater/measurement ICC were calculated. Since ICC values achieved were very high, average data from both assessments was used in further analysis.

Data are presented as n (% of group) for nominal variables or as mean ± SD or median (Q1; Q3) for continuous variables, as appropriate. Normality of distribution was assessed using Shapiro–Wilk test as well as based on skewness and kurtosis values. Group comparisons was based on tests: Fisher’s exact test or chi-square test for nominal data, t-test or Mann–Whitney U test for continuous data, as appropriate. Additionally, MD (mean/median difference) between both groups as well as OR (odds ratio) were calculated, including 95% confidence interval (CI). Correlation between parameters was assessed using Pearson’s correlation coefficient. Correlation analyses were exploratory.

Univariate linear regression analysis was carried out to determine relationship between the primary endpoint (pupil diameter) and selected clinical/systemic variables in SSc (e.g., disease duration, subtype, autoantibodies, vasoactive therapy, mean arterial pressure MAP, smoking). Analysis of covariance (ANCOVA) was conducted with group status (SSc vs. controls) as the independent variable and MAP as a prespecified covariate. Adjusted means, expressed as estimated marginal means with 95% confidence intervals, were calculated for both groups. Additional exploratory regression models were performed for secondary endpoints (iris cross-sectional area/thickness) as sensitivity analyses. Sample size power determination was made using G*Power 3.1.9.7. software. Sample size power (1-beta value) was assessed post-hoc based on the data achieved in the study and assuming *α* = 0.05 for parameters: pupil diameter, T Iris cross-sectional area, N Iris cross-sectional area, and amounted to values: 0.57, 0.06, and 0.06, respectively.

## 3 Results

A total of 63 participants were enrolled in the study, comprising 31 patients with SSc and 32 healthy controls. Baseline demographic and ophthalmological characteristics are presented in Table 2, and disease-specific clinical characteristics of patients with SSc are summarized in Table 3. There were no significant differences between groups in age, sex, axial length, intraocular pressure, or iris color. Mean arterial pressure (MAP) was significantly lower in patients with SSc than in controls (*p* = 0.001). The majority of patients had diffuse cutaneous SSc and active or late nailfold capillaroscopy patterns, reflecting established microvascular involvement. More than half of the cohort was anti–Scl-70 positive, and pulmonary involvement was common, while a substantial proportion of patients received vasoactive therapies, including phosphodiesterase inhibitors and calcium channel blockers.

**Table 2:** Baseline demographic and ophthalmological characteristics of patients with systemic sclerosis and healthy controls.

Variable	SSc group	Control group	MD/OR	95% CI for MD/OR	*p*
Age, mean±SD, years	51.39 ± 12.53	49.41 ± 10.79	1.98	−3.92;7.89	0.505
Sex, *n* (%)					
Female	22 (71.0)	18 (56.3)	0.53	0.16; 1.68	0.341
Male	9 (29.0)	14 (43.8)			
Nicotine, *n* (%), yes	3 (9.7)	6 (18.8)	0.47	0.07;2.48	0.474^2^
MAP, mean±SD, mmHg	86.60 ± 9.56	96.60 ± 11.24	−10.01	−15.73; −4.28	**0.001**
AXL, mean±SD, mm	23.22 ± 0.82	23.35 ± 0.89	−0.12	−0.55; 0.31	0.570
logMAR, median (Q1; Q3) / mean±SD	0.00 (0.00;0.00) / 0.02 ± 0.06	0.00 (0.00;0.00) / 0.01 ± 0.02	0.00	−0.01; 0.01	0.935^1^
IOP, mean±SD, mmHg	13.98 ± 3.13	15.04 ± 2.03	−1.06	−2.56; 0.44	0.160
Iris color, *n* (%)					
green or blue	20 (66.7)	24 (75.0)	0.67	0.19;2.30	0.658
brown/ brown-green/ green-brown	10 (33.3)	8 (25.0)			
SE, mean±SD, D	0.69 ± 1.09	0.41 ± 1.50	0.28	−0.38;0.94	0.400
K1, mean±SD, mm	7.82 ± 0.26	7.86 ± 0.28	−0.04	−0.17;0.10	0.594
K2, mean±SD, mm	7.67 ± 0.29	7.72 ± 0.29	−0.05	−0.19;0.10	0.513

*groups compared with independent t-test or Mann–Whitney U test^1^ for continuous variables and with chi-square test or Fisher exact test^2^ for nominal variables. AXL, Axial length; CI, Confidence interval; D, Diopters; IOP, Intraocular pressure; K1, Flat keratometry; K2, Steep keratometry; MAP, Mean arterial pressure; MD, Mean/median difference between groups calculated as SSc group minus Control group; OR, Odds ratio; Q1, Lower quartile; Q3, Upper quartile; SE, spherical equivalent; SSc, Systemic sclerosis, *p* < 0.05 in bold.

**Table 3:** Disease-specific clinical characteristics of patients with systemic sclerosis.

Domain	Parameter	SSc group (*n* = 31)
Disease subtype/microvascular involvement NFC, Cutolo classification (15), *n* (%)	dSSc	21 (67.7)
	lSSc	10 (32.3)
	Active	15 (48.4)
	Early	8 (25.8)
	Late	8 (25.8)
Disease duration	Duration (years), median (Q1; Q3)	4.00 (2.00; 10.50)
Autoantibody profile, *n* (%)	Anti-Scl-70	15 (53.6)
	Anti-centromere	6 (21.4)
	Anti-CCP	1 (3.6)
	Other antibodies	13 (46.4)
Organ involvement	Joint involvement, n (%)	15 (48.4)
	ILD/fibrosis (HRCT), n (%)	22 (71.0)
	CRP (mg/L), median (Q1; Q3)	1.45 (1.00; 3.23)
	Cardiac involvement, n (%)	10 (32.3)
	Digital ulcers (past or present), n (%)	10 (32.3)
Systemic treatment	PDE5 inhibitors, n (%)	15 (48.4)
	Calcium-channel blockers, n (%)	8 (25.8)
	Hydroxychloroquine, n (%)	6 (19.4)
	Steroids, n (%)	12 (38.7)
	Diuretics, n (%)	9 (29.0)

Abs, antibodies; anti-CCP, anti–cyclic citrullinated peptide antibodies; anti-Scl-70, anti–topoisomerase I antibodies; CRP, C-reactive protein; dSSc, diffuse cutaneous systemic sclerosis; HRCT, High-resolution computed tomography; ILD, Interstitial Lung Disease; lSSc, Limited cutaneous systemic sclerosis; MAP, Mean arterial pressure; NFC, Nailfold capillaroscopy; PDE, Phosphodiesterase; Q1, Lower quartile; Q3, Upper quartile; SSc, Systemic sclerosis.

### 3.1 Baseline characteristics

Baseline demographic, ophthalmological, and systemic characteristics of the study participants are summarized in Tables 2, 3. Table 2 presents comparative baseline data between patients with SSc and healthy controls, whereas Table 3 details disease-specific clinical features, organ involvement, and treatment characteristics within the SSc cohort.

### 3.2 Intergroup comparisons of iris thickness, cross-sectional area, and OCT-derived pupil diameter

Intergroup comparisons of AS-OCT parameters are presented in Table 4. Pupil diameter was significantly smaller in the SSc group (*p* = 0.033). Due to the statistically significant difference in MAP between groups, an additional sensitivity analysis was performed for the primary outcome. In ANCOVA, the direction and magnitude of the association remained similar, with lower pupil diameter observed in the SSc group (*β* = −366.9 μm, *p* = 0.057). However, MAP was not significantly associated with pupil diameter in the adjusted model (*p* = 0.636). Adjusted mean pupil diameter was 3,547 μm (95% CI: 3,288–3,807) in controls and 3,180 μm (95% CI: 2,931–3,430) in patients with SSc. The overall model fit was limited (*R*^2^ = 0.07). Iris thickness measured at 1, 2, and 3 mm from the pupil margin, as well as nasal and temporal iris cross-sectional areas, did not differ significantly between patients with SSc and healthy controls.

**Table 4:** Comparison of OCT-derived pupil diameter, iris thickness, and iris cross-sectional area between patients with systemic sclerosis and healthy controls.

Parameter		SSc group (mean±SD)	Control group (mean±SD)	MD	95% CI for MD	*p*
Pupil diameter [μm]		3170.24 ± 533.26	3518.18 ± 720.86	−347.93	−667.14; −28.73	**0.033**
Iris thickness [μm]	1 mm T	438.57 ± 78.61	436.92 ± 73.64	1.66	−36.75; 40.06	0.932
	2 mm T	397.75 ± 89.27	402.20 ± 86.44	−4.45	−48.75; 39.84	0.841
	3 mm T	346.24 ± 81.46	369.02 ± 75.67	−22.78	−62.43; 16.87	0.255
	1 mm N	519.06 ± 91.24	492.97 ± 85.07	26.09	−18.39; 70.57	0.245
	2 mm N	421.72 ± 62.93	438.56 ± 85.19	−16.84	−54.54; 20.87	0.375
	3 mm N	355.97 ± 66.79	376.16 ± 68.12	−20.19	−54.18; 13.79	0.240
Iris cross-sectional area [μm^2^]	T	1095919.85 ± 167022.07	1108019.56 ± 165955.84	−12099.71	−96004.27; 71804.85	0.774
	N	1227348.78 ± 152526.70	1240747.79 ± 169197.04	−13399.01	−94511.19; 67713.17	0.742

CI, Confidence interval; MD, Mean difference between groups calculated as SSc group minus Control group; groups compared with independent *t*-test. *iris thickness measured without iris pigment epithelium. *iris cross-sectional area measured up to 3 mm from pupil margin. N, nasal; T,temporal; 1/2/3 mm, distance from pupil margin, *p* < 0.05 in bold.

A sensitivity sub-analysis comparing static AS-OCT pupil diameter between SSc patients receiving and not receiving vasoactive treatment was performed. Subgroup comparisons showed no significant difference in pupil diameter between SSc patients receiving and not receiving PDE5 inhibitors (3,107.8 ± 639.8 μm vs. 3,227.2 ± 408.8 μm, *p* = 0.46), nor between those receiving and not receiving calcium-channel blockers (2,943.6 ± 579.7 μm vs. 3,247.9 ± 497.5 μm, *p* = 0.21), with treated patients showing numerically smaller rather than larger pupils.

### 3.3 Intra-group correlations between OCT-derived pupil diameter and ocular parameters

Correlations between pupil diameter and ocular structural parameters are shown in Table 5. In the SSc group, no significant associations were observed between pupil diameter and choroidal or iris parameters. In contrast, in the control group, pupil diameter correlated positively with nasal iris cross-sectional area measured 3 mm from the pupil margin and with iris thickness measured 1 mm from the pupil margin.

**Table 5:** Correlations between pupil diameter and ocular structural parameters.

Parameter		SSc group		Control group	
		*r*	*p*	*r*	*p*
Iris cross-sectional area [μm^2^]	T	−0.08	0.675	0.29	0.111
	N	−0.14	0.446	0.43	**0.013**
Iris thickness [μm]	1 mm T	0.11	0.574	0.47	**0.007**
	2 mm T	0.03	0.890	0.01	0.943
	3 mm T	0.18	0.337	0.12	0.522
	1 mm N	−0.27	0.146	0.55	**0.001**
	2 mm N	0.07	0.711	0.09	0.630
	3 mm N	0.17	0.364	0.12	0.496
	mCVI (%)	0.16	0.433	0.09	0.629
	Choroidal thickness central macular (μm)	0.16	0.429	0.04	0.831
	SFCT (μm)	0.17	0.407	0.03	0.881
	Choroidal volume central macular (mm^3^)	0.14	0.486	0.05	0.799
	Choroidal volume total (mm^3^)	0.10	0.628	0.08	0.666
	mTCA (μm^2^)	0.16	0.442	−0.05	0.802
	mCVI (%)	0.16	0.433	0.09	0.629
	pTCA (μm^2^)	0.14	0.472	0.03	0.854
	pCVI (%)	0.22	0.247	−0.17	0.360
	pCT Global (μm)	0.08	0.675	−0.05	0.784

*iris cross-sectional area measured up to 3 mm from pupil margin. *iris thickness measured without iris pigment epithelium. m, macular; mCVI, macular choroidal vascularity index; mTCA, macular total choroidal area; N, nasal; p, peripapillary; pCVI, Peripapillary choroidal vascularity index; pCT global, Peripapillary choroidal thickness; pTCA, Peripapillary total choroidal area; r, Pearson correlation coefficient; SFCT, Subfoveal choroidal thickness; SSc, Systemic sclerosis; T, Temporal; 1/2/3 mm, distance from pupil margin, *p* < 0.05 in bold.

### 3.4 Intra-group univariate regressions to identify factors associated with OCT-derived pupil diameter

Univariate regression analyses for pupil diameter in the SSc cohort are presented in Table 6. Among the tested demographic, clinical, and ocular variables, smoking status showed a significant association with smaller pupil diameter, whereas age, sex, axial length, mean arterial pressure, and disease-related characteristics were not significantly associated. Given the exploratory nature of the analysis and the very small number of smokers in the SSc group (n = 3), this finding should be interpreted with caution.

**Table 6:** Univariate linear regression analysis of factors associated with pupil diameter in patients with systemic sclerosis.

Domain	Parameter	*β*	Standard error	B	*p*	*R* ^2^	adj *R*^2^
Demographic and clinical parameters	Age (years)	−8.005	7.759	−0.188	0.311	0.035	0.002
	Sex, male	78.150	214.120	–	0.718	0.005	−0.029
	Nicotine, yes	−801.290	293.980	–	**0.011**	0.204	0.177
	MAP (mmHg)	−6.049	10.377	−0.108	0.565	0.013	−0.025
	Disease duration (years)	−3.476	13.060	−0.005	0.792	0.002	−0.032
Ophthalmological parameters	AXL (mm)	77.410	119.640	0.119	0.523	0.014	−0.019
	logMAR	23.160	1553.220	0.003	0.988	<0.001	−0.034
	IOP (mmHg)	8.119	29.357	0.048	0.785	0.003	−0.041
	Iris color (brown/brown-green/green-brown)	283.500	206.800	–	0.181	0.063	0.029
Disease subtype/microvascular involvement NFC, Cutolo classification (15), *n* (%)	dSSc vs. lSSc	24.700	208.300	–	0.906	0.001	−0.034
	Early vs. Active	−50.240	240.950	–	0.836	0.006	−0.065
	Late vs. Active	−95.580	240.950	–	0.695	–	–
Autoantibody profile	anti-Scl-70, yes	−11.020	208.530	–	0.958	0.000	−0.039
	anti-centromere, yes	−270.800	247.800	–	0.285	0.044	0.007
	anti-CCP, yes	−70.940	560.270	–	0.900	0.001	−0.038
	Other Abs, yes	−20.430	208.510	–	0.923	0.000	−0.038
Organ involvement	Joint involvement, yes	−104.100	194.000	–	0.596	0.009	−0.024
	Pulmonary involvement (ILD/fibrosis)	100.300	213.800	–	0.643	0.008	−0.027
	Cardiac involvement, yes	−318.400	199.800	–	0.122	0.080	0.049
	Digital ulcers, yes	−74.420	207.930	–	0.723	0.004	−0.030
Inflammation	CRP (mg/L)	−6.011	50.419	−0.024	0.906	0.001	−0.039
Systemic treatment	PDE5 inhibitors, yes	−122.800	193.600	–	0.531	0.014	−0.020
	Calcium-channel blockers, yes	−306.100	215.200	–	0.166	0.065	0.033
	Hydroxychloroquine, yes	−376.200	236.500	–	0.122	0.080	0.049
	Steroids, yes	53.410	199.750	–	0.791	0.002	−0.032
	Diuretics, yes	−372.600	203.200	–	0.077	0.104	0.073
Choroidal parameters	mTCA (μm^2^)	0.001	0.002	0.152	0.442	0.025	−0.016
	mCVI (%)	32.280	40.460	0.158	0.433	0.027	−0.015
	pTCA (μm^2^)	0.000	0.000	0.137	0.472	0.019	−0.016
	pCVI (%)	63.350	53.620	0.219	0.247	0.047	0.013
	pCT Global (μm)	0.719	1.699	0.078	0.675	0.006	−0.028
	Choroidal thickness central macular (μm)	1.265	1.575	0.159	0.429	0.025	−0.014
	SFCT (μm)	1.259	1.493	0.167	0.407	0.028	−0.011
	Choroidal volume central macular (mm^3^)	1443.400	2038.900	0.141	0.486	0.020	−0.020
	Choroidal volume total (mm^3^)	35.230	71.880	0.097	0.628	0.009	−0.030
Iris cross-sectional area	Temporal	0.000	0.001	0.081	0.675	0.006	−0.029
	Nasal	−0.001	0.001	−0.142	0.446	0.020	−0.014
Iris thickness	1 mm T	0.745	1.309	0.099	0.574	0.011	−0.024
	2 mm T	0.214	1.530	0.028	0.890	0.001	−0.036
	3 mm T	1.352	1.385	0.183	0.337	0.033	−0.002
	1 mm N	−1.564	1.046	−0.267	0.146	0.072	0.040
	2 mm N	0.587	1.570	0.069	0.711	0.005	−0.030
	3 mm N	1.347	1.461	0.169	0.364	0.028	−0.005

β, beta coefficient; B, Standardized beta. *iris cross-sectional area measured up to 3 mm from pupil margin. *iris thickness measured without iris pigment epithelium. Abs, antibodies; AXL, axial length; anti-CCP, anti–cyclic citrullinated peptide antibodies; anti-Scl-70, anti–topoisomerase I antibodies; CRP, C-reactive protein; dSSc, diffuse cutaneous systemic sclerosis; HRCT, High-resolution computed tomography; ILD, Interstitial lung disease; IOP, Intraocular pressure; lSSc, Limited cutaneous systemic sclerosis; MAP, Mean arterial pressure; mCVI, Macular choroidal vascularity index; mTCA, Macular total choroidal area; N, nasal; NFC, Nailfold capillaroscopy; pCVI, Peripapillary choroidal vascularity index; pCT global, Peripapillary choroidal thickness; pTCA, Peripapillary total choroidal area; SFCT, Subfoveal choroidal thickness; SSc, Systemic sclerosis; T, Temporal, 1/2/3 mm- distance from the pupil margin, *p* < 0.05 in bold.

Univariate regression analyses for pupil diameter in the control group are summarized in Table 7. In healthy controls, pupil diameter showed a significant negative association with age. In addition, nasal iris cross-sectional area demonstrated a modest but significant relationship with pupil diameter in controls (*p* = 0.013; Supplementary Table S2).

**Table 7:** Univariate linear regression analysis of factors associated with pupil diameter in the control group.

Pupil diameter (μm) vs. following variables:	Parameter	*β*	Standard error	B	*p*	adj *R*^2^
Demographic and clinical parameters	Age (years)	−37.290	10.110	−0.558	**0.001**	0.289
	Sex, male	176.100	259.100	–	0.502	−0.018
	Nicotine, yes	−158.500	330.600	–	0.635	−0.026
	MAP (mmHg)	0.571	17.636	0.006	0.975	−0.045
Ophthalmological parameters	AXL (mm)	190.800	143.700	0.236	0.194	0.024
	logMAR	5354.500	5261.400	0.183	0.317	0.001
	IOP (mmHg)	44.420	69.840	0.125	0.530	−0.022
	Iris color, brown/brown-green/ green-brown	132.900	298.200	–	0.659	−0.027
Choroidal parameters	mTCA (μm^2^)	0.000	0.002	−0.047	0.802	−0.033
	mCVI (%)	28.610	58.480	0.090	0.628	−0.027
	pTCA (μm^2^)	0.000	0.000	0.034	0.854	−0.032
	pCVI (%)	−62.410	67.080	−0.168	0.360	−0.004
	pCT Global (μm)	−0.794	2.872	−0.051	0.784	−0.032
	Choroidal thickness central macular (μm)	0.385	1.784	0.039	0.830	−0.032
	SFCT (μm)	0.239	1.584	0.028	0.881	−0.033
	Choroidal volume central macular (mm^3^)	570.100	2216.900	0.047	0.799	−0.031
	Choroidal volume total (mm^3^)	44.710	102.410	0.082	0.666	−0.028
Iris cross-sectional area [μm^2^]	T	0.001	0.001	0.287	0.111	0.052
	N	0.002	0.001	0.434	**0.013**	0.162
Iris thickness [μm]	1 mm T	4.609	1.577	0.471	**0.007**	0.196
	2 mm T	0.215	2.970	0.015	0.943	−0.038
	3 mm T	1.120	1.727	0.118	0.522	−0.019
	1 mm N	4.663	1.292	0.550	**0.001**	0.279
	2 mm N	0.748	1.539	0.088	0.630	−0.025
	3 mm N	1.320	1.917	0.125	0.496	−0.017

β, beta coefficient; B, standardized beta. *iris cross-sectional area measured up to 3 mm from pupil margin. *iris thickness measured without iris pigment epithelium. AXL, axial length; IOP, Intraocular pressure; lSSc, Limited cutaneous systemic sclerosis; MAP, Mean arterial pressure; mCVI, Macular choroidal vascularity index; mTCA, Macular total choroidal area; N, Nasal; pCVI, Peripapillary choroidal vascularity index; pCT global, Peripapillary choroidal thickness; pTCA, Peripapillary total choroidal area; SFCT, Subfoveal choroidal thickness; T, Temporal, 1/2/3 mm- distance from pupil margin, *p* < 0.05 in bold.

### 3.5 Exploratory supplementary analyses

Exploratory analyses presented in the Supplement (Supplementary Tables S1–S6) focused on iris cross-sectional area (nasal and temporal, measured up to 3 mm from the pupil margin) rather than point-based iris thickness, because cross-sectional area provides a more global descriptor of iris morphology and is less dependent on a single measurement location. Given the number of variables tested and the small subgroups, these nominal associations should be regarded as hypothesis-generating only. The condensed Supplementary Tables S3–S6 are provided for completeness.

## 4 Discussion

This cross-sectional OCT study provides a quantitative assessment of static AS-OCT-derived pupil diameter, iris thickness, and iris cross-sectional area in patients with SSc compared with healthy controls. Using ImageJ-based quantification of standardized OCT scans, we found no significant differences in iris thickness (at 1, 2, and 3 mm from the pupil margin) or in nasal/temporal iris cross-sectional area (up to 3 mm from the pupil margin). In contrast, pupil diameter was smaller in the SSc group under identical acquisition conditions. Given the limited sample size, the cross-sectional design, and the use of static OCT-derived pupil measurement rather than dynamic pupillometry, this finding should be considered hypothesis-generating and requires confirmation in larger cohorts.

Pupil diameter is primarily governed by the autonomic nervous system, which consists of two opposing divisions: the sympathetic and parasympathetic systems. The dynamic balance between these branches regulates pupillary size in response to light intensity, emotional arousal, and other internal or external stimuli. Sympathetic activation leads to mydriasis, whereas parasympathetic stimulation results in miosis (29). Neurological involvement in SSc affects both the central and peripheral nervous systems. Peripheral nervous system manifestations are relatively common, occurring in approximately 16–35% of cases (30), and may include cranial nerve dysfunction as well as spinal nerve abnormalities such as motor and sensory polyneuropathies. Raynaud’s phenomenon occurs in 90 to over 95% of patients with SSc (31, 32). In its pathogenesis, studies have demonstrated increased sympathetic activity accompanied by decreased parasympathetic activity. This autonomic imbalance contributes to exaggerated vasoconstriction of peripheral blood vessels in response to cold or emotional stress, which is characteristic of Raynaud’s episodes. The dominance of sympathetic vasomotor tone leads to reduced blood flow and ischemia in affected body parts (30, 33). Del Rosso et al. investigated autonomic nervous system involvement in SSc by assessing baseline pupil diameter and pupillary response to substance P using pupillometry. Substance P is a neuropeptide that induces miosis via stimulation of the sphincter pupillae muscle. Patients with SSc exhibited a significantly reduced resting pupil diameter and a pronounced constrictive response compared to controls. These findings indicate peripheral autonomic dysfunction in SSc, with more pronounced impairment in lcSSc (29). Pupillometry remains the reference method for detailed assessment of pupil dynamics. It is used to assess autonomic function by quantifying dynamic pupil responses (baseline diameter, constriction amplitude/latency, and redilation). These measures offer functional insight into sympathetic-parasympathetic balance but require dedicated equipment and tightly controlled testing conditions (38). In contrast, AS-OCT provides rapid, non-invasive structural imaging during routine examination, from which pupil diameter can be extracted as a static parameter alongside iris morphometry without additional equipment or examination burden. Although OCT does not capture dynamic autonomic responses, OCT-derived pupil diameter could be considered a complementary, broadly accessible metric that may serve as a hypothesis-generating marker of neurovascular involvement in SSc. However, it should not be interpreted as a substitute for dynamic pupillometry.

A smaller static AS-OCT-derived pupil diameter in SSc is biologically plausible in the context of previously reported autonomic involvement in this disease. Pupil diameter reflects the balance between sympathetic and parasympathetic inputs, and autonomic dysfunction has been described in SSc, including within the broader context of Raynaud-related neurovascular dysregulation. Prior pupillometry studies reporting reduced baseline pupil diameter and altered pupillary responses in SSc support peripheral autonomic impairment as a potential mechanism (29–33). However, because our study assessed pupil diameter from static AS-OCT images rather than dynamic pupillometry, this finding should be interpreted cautiously. It may be consistent with, but does not directly demonstrate, autonomic or neurovascular dysfunction. In addition, since our results are based on specific room conditions, varying clinical environments may result in different absolute pupil diameter.

Interestingly, intra-group analyses suggested that in controls, pupil diameter correlated with selected anterior iris parameters (nasal iris cross-sectional area and iris thickness at 1 mm nasally and temporally), whereas such relationships were not observed in SSc. This pattern likely reflects physiological coupling between pupil size and iris configuration in healthy eyes, with a potential decoupling in SSc related to altered microvasculature and neurovascular regulation, medication effects, or residual variability in pupil-related conditions during acquisition.

In healthy controls, pupil diameter showed the expected age-related decline (39). In contrast, this relationship was not observed in the SSc cohort. This discrepancy does not necessarily indicate an absence of age-related effects in SSc (autonomic dysregulation, vasoactive therapies, microvascular involvement) but rather suggests that disease-related factors may override or mask the physiological influence of age on pupil size.

The absence of detectable differences in iris thickness and iris cross-sectional area does not necessarily exclude anterior uveal involvement in SSc. First, our segmentation approach deliberately excluded posterior epithelial layers (iris pigment epithelium) due to limited OCT penetration in highly pigmented tissue and blurred posterior borders, which improves edge reliability but may reduce sensitivity to disease-related changes that preferentially affect posterior or pigment-related structures. Accordingly, our protocol may be less sensitive to pigment epithelium-related abnormalities (e.g., transillumination defects) reported in SSc. Second, iris measurements were derived from a single horizontal scan centered on the pupil; if SSc-related abnormalities are regional or heterogeneous, a single meridian may miss focal effects. Third, methodology differs across studies, including reference landmarks (pupil margin versus scleral spur), which limits direct comparison. These considerations support interpreting the negative iris-thickness/cross-sectional area findings as no difference detected with this protocol rather than definitive absence of iris changes in SSc.

The iris and choroid are components of the uvea, and several conditions (e.g., pseudoexfoliation syndrome, Fuchs uveitis) have been reported to affect their thickness (24, 40, 41). These disorders were not present in our participants. Kaya et al. demonstrated significant associations between iris thickness and choroidal thickness at selected locations in healthy subjects (42). Using ultrasound biomicroscopy (UBM), Yousef et al. reported that nasal iris thickness exceeds temporal thickness in a Middle Eastern population, consistent with our findings, although methodological differences (UBM vs. AS-OCT and exclusion of posterior epithelial layers in our study) limit direct quantitative comparisons (43). Similar nasal-temporal asymmetry was also observed by Garcia et al. using UBM, with nasal segments significantly thicker than temporal segments (44).

Key limitations include potential residual variability in pupil diameter driven by unmeasured factors (caffeine/nicotine timing, emotional arousal), use of a single scan meridian, and limited statistical power for some outcomes. A major limitation of the present study is that pupil diameter was assessed statically from AS-OCT images rather than with dedicated dynamic pupillometry. Consequently, we could not evaluate pupillary light reflex parameters, which would provide a more direct assessment of autonomic function. Although minor image tilt cannot be fully excluded, its potential impact is minimized by strict image quality criteria, dual-axis calibration, local perpendicular measurements, and area-based segmentation. A further limitation is pupillary hippus, the continuous physiological oscillation of pupil diameter that occurs even under constant illumination. Although we standardized illuminance, light-adaptation time, fixation, and time of day to minimize this effect, a single static scan cannot eliminate random measurement noise. Because hippus introduces moment-to-moment variability that is not captured by a single acquisition, part of the observed between-group difference and within-group scatter may reflect this fluctuation rather than a stable trait. Furthermore, we did not assess within session or between visit repeatability of pupil diameter and iris metrics; therefore, biological variability of these parameters in SSc could not be quantified in this study. *Post hoc* power estimates were low for iris cross-sectional areas and suboptimal for pupil diameter, suggesting the study may be underpowered to detect small to moderate differences and that the significant pupil finding should be confirmed. Finally, the small sample size precluded robust multivariable modeling incorporating several covariates simultaneously. Given the number of tested parameters and exploratory regressions, the pupil result warrants cautious interpretation. The absence of significant structural iris differences should be interpreted as no detectable difference using the present protocol and sample size, rather than as definitive evidence of no anterior uveal involvement in SSc. The nominal associations for iris cross-sectional area were exploratory, unadjusted for multiple comparisons, and based on small subgroups; therefore, they should be considered a basis for further investigation rather than confirmatory. Clinical and systemic factors may confound or mediate pupil diameter and uveal measurements in SSc.

In our cohort, MAP was lower in SSc than in controls, and a substantial proportion of SSc patients received vasoactive therapies, including phosphodiesterase inhibitors and calcium-channel blockers. These factors may potentially influence systemic hemodynamics and neurovascular tone and should therefore be considered possible confounders rather than established explanations for the observed difference in pupil diameter. To address the difference in MAP, we performed a limited sensitivity analysis for the primary outcome, adjusting for MAP. The direction and magnitude of the association between SSc status and smaller static pupil diameter remained similar after adjustment, although the adjusted group effect did not reach conventional statistical significance. MAP itself was not significantly associated with pupil diameter in the adjusted model, and the overall model fit was limited, supporting cautious interpretation. For both drug classes the point estimates were comparable, and treated patients had a numerically smaller rather than larger pupil diameter, contrary to a vasodilatory or mydriatic effect. Thus, within this cohort, vasoactive medication exposure did not explain the smaller pupil diameter observed in SSc relative to controls. This is consistent with available evidence, which does not consistently support a clinically relevant miotic effect of phosphodiesterase-5 inhibitors or calcium-channel blockers that would explain smaller pupil diameter in SSc, including reports of mydriasis rather than miosis with calcium-channel blockers (45–47). Nevertheless, residual confounding by systemic hemodynamic status, vasoactive medication exposure, or other unmeasured factors cannot be excluded.

This study has several strengths: standardized OCT acquisition within a restricted time window (to reduce diurnal variation), imaging performed by the same experienced examiner, and excellent inter-rater agreement across all iris measurements, supporting measurement robustness. In addition, we used a pragmatic acquisition workflow that can be implemented in routine practice (single horizontal OCT B-scan), while still applying uniform illumination/dark adaptation procedures and consistent image quality thresholds. Quantification in ImageJ enabled evaluation of both point-based thickness and a more global cross-sectional area measure, which is less dependent on a single local landmark and may better capture overall iris morphology in the presence of crypts and surface undulations. Masking of graders to clinical data and averaging of two independent measurements with near perfect ICC values further reduces the likelihood that subtle group differences are driven by measurement noise. We also minimized key confounders by excluding participants using medications known to directly affect pupil diameter and by accounting for iris pigmentation categories. Finally, the availability of detailed systemic phenotyping in the SSc cohort (subtype, autoantibodies, nailfold capillaroscopy patterns, organ involvement, and vasoactive therapy exposure) provides a foundation for future multivariable analyses in larger samples aimed at linking anterior segment metrics with systemic microvascular disease features.

Future work should validate these findings in adequately powered, preferably multicenter cohorts with prespecified primary outcomes and improved control of pupil-related acquisition variables. Incorporating dynamic pupillometry (baseline diameter and light reflex parameters), repeated measurements (test–retest), complementary anterior segment vascular imaging (e.g., AS-OCTA where available), and validated automated software for mathematical image straightening, geometric rectification, and quantitative analysis of iris B-scans would strengthen the interpretation and potentially link ocular metrics with systemic microvascular phenotypes (e.g., nailfold capillaroscopy patterns) and disease subtypes. Future studies could clarify whether pupil diameter is a reproducible ocular feature of SSc-related autonomic involvement and whether iris morphology changes emerge with different imaging strategies, landmarks, or inclusion of pigment-related layers when technically feasible. From a translational perspective, OCT-derived pupil diameter may be incorporated into routine anterior segment imaging workflows in patients with SSc without imposing an additional examination burden. This simple parameter may serve as a screening-level indicator of systemic neurovascular involvement, complementing posterior segment oculomic markers such as choroidal thickness and vascularity index.

## 5 Conclusion

In patients with SSc, AS-OCT revealed no differences in iris thickness or iris cross-sectional area compared with healthy controls, whereas static OCT-derived pupil diameter was smaller under standardized acquisition conditions. This finding, observed in the absence of structural iris changes, may reflect functional rather than morphological alterations of the anterior uveal tract. Given the limited sample size and exploratory character of the study, OCT-derived pupil diameter should be considered a preliminary, hypothesis-generating parameter that warrants validation in adequately powered cohorts, preferably with dynamic pupillometry.

## Data Availability

The raw data supporting the conclusions of this article will be made available by the authors, without undue reservation.

## Footnotes

1 https://imagej.net/ij/, ImageJ 1.54 g, NIH, USA; accessed 18 November 2023.
