# Supplementary material for: Anterior segment oculomics in systemic sclerosis: reduced OCT-derived pupil diameter without iris morphology alterations
Source: Front Med (Lausanne). 2026 Aug 7;13:1776963. doi: 10.3389/fmed.2026.1776963 (PMC13493390; doi:10.3389/fmed.2026.1776963)
Supplement: Supplementary file 1 [file Supplementary_file_1.docx]

Supplementary Material - Step-by-Step Measurement Methodology

**Title: Anterior segment oculomics in systemic sclerosis: reduced OCT-derived pupil diameter without iris morphology alterations**

1 **Image acquisition**

A single horizontal anterior segment (AS)-OCT B-scan (30°, Heidelberg Engineering GmbH, Heidelberg, Germany, 2016), centered on the pupil, was exported as an image and opened in ImageJ software (NIH, USA, <https://imagej.net/ij/>).

2 **Scale calibration**
  2.1 **Horizontal calibration**

The image was enlarged for accurate calibration. Using the Line tool, the pixel length corresponding to 200 µm was measured based on the horizontal scale bar displayed at the bottom of the OCT image. This value was entered in Analyze → Set Scale, assigning the unit as µm.

2.2 **Vertical calibration**

The calibration was cleared (Analyze → Set Scale → Click to remove scale) and the vertical scale was calibrated analogously using the corresponding scale reference in the OCT image.

2.3 **Pixel aspect ratio**

The pixel aspect ratio was calculated as the ratio of horizontal to vertical pixel size (e.g., 17/17 = 1.0). The horizontal scale was then entered again, and the verified pixel aspect ratio (1.0) was applied in the pixel aspect ratio field. The unit of length was set to micrometers, and the Global option was selected for subsequent measurements.

3 **Pupil diameter**

Pupil diameter was measured using the Line tool between the two visible pupil margins.

4 **Iris measurements**

The methodology is first described for the nasal iris measurements:

a) A distance of 3,000 µm was measured using the line tool, from the nasal pupil margin in a straight line perpendicular to the iris pigment epithelium.

b) In the same manner, distances of 2,000 µm and 1,000 µm were marked along a straight line toward the nasal pupil margin.

c) Iris thickness: At each marked location (1,000, 2,000, and 3,000 µm), a line perpendicular to the local iris surface was constructed (using the Angle/Protractor tool to set 90°). Iris thickness was measured as the distance from the anterior iris surface to the anterior border of the iris pigment epithelium using the Line tool.

d) Nasal iris cross-sectional area: was measured using the Polygon tool by delineating the iris region from the nasal pupil margin toward the line representing iris thickness at a distance of 3,000 µm from the nasal pupil margin (bounded by the anterior iris surface and anterior surface of the iris pigment epithelium).

e) Iris thickness at 1,000/2,000/3,000 µm and iris cross-sectional area were then measured on the temporal side using the same procedure.
