# Supplementary material for: Anterior segment oculomics in systemic sclerosis: reduced OCT-derived pupil diameter without iris morphology alterations
Source: Front Med (Lausanne). 2026 Aug 7;13:1776963. doi: 10.3389/fmed.2026.1776963 (PMC13493390; doi:10.3389/fmed.2026.1776963)
Supplement: Supplementary file 2 [file Supplementary_file_2.docx]

Supplementary Tables S1-S6

**Title: Anterior segment oculomics in systemic sclerosis: reduced OCT-derived pupil diameter without iris morphology alterations**

*Choroidal parameters were previously published in:

a) Pieklarz B, Gińdzieńska-Sieśkiewicz E, Zawadzka I, Bagrowska M, Daniluk J, Palewski M, et al. Macular choroidal thickness, volume, and vascularity index in patients with systemic sclerosis. Graefes Arch Clin Exp Ophthalmol. 2024;262:1475-87.
b) Pieklarz B, Gińdzieńska-Sieśkiewicz E, Zawadzka I, Bagrowska M, Daniluk J, Sidorczuk P, et al. Peripapillary choroidal vascularity index and thickness in patients with systemic sclerosis. Front Med (Lausanne). 2023;10:1273438.
1 **Intra-group correlations between iris cross-sectional area and pupil/choroidal parameters.**

**Table S1.** Correlations between temporal iris cross-sectional area and pupil/choroidal parameters.

| **T Iris cross-sectional area (µm²) measured 3 mm from the pupil margin and the following parameters** | **SSc group** | | **Control group** | |
| --- | --- | --- | --- | --- |
|  | r | p | r | p |
| **Pupil diameter (µm)** | -0.08 | 0.675 | 0.29 | 0.111 |
| **mTCA (µm²)** | -0.21 | 0.311 | 0.2 | 0.299 |
| **mCVI (%)** | 0.19 | 0.376 | 0.26 | 0.159 |
| **pTCA (µm²)** | -0.04 | 0.821 | 0.16 | 0.394 |
| **pCVI (%)** | -0.24 | 0.211 | 0.08 | 0.649 |
| **pCT Global (µm)** | -0.08 | 0.662 | 0.11 | 0.562 |
| **Choroidal thickness central macular (µm)** | -0.06 | 0.765 | 0.18 | 0.321 |
| **SFCT (µm)** | -0.07 | 0.734 | 0.17 | 0.339 |
| **Choroidal volume central macular (mm³)** | -0.04 | 0.831 | 0.18 | 0.337 |
| **Choroidal volume total (mm³)** | 0.09 | 0.648 | 0.19 | 0.314 |

r – Pearson correlation coefficient

Abbreviations: mCVI – macular choroidal vascularity index, mTCA – macular total choroidal area, pCVI – peripapillary choroidal vascularity index, pCT global – peripapillary choroidal thickness, pTCA – peripapillary total choroidal area, r – Pearson correlation coefficient, SFCT – subfoveal choroidal thickness, SSc – systemic sclerosis, p<0.05 in bold

**Table S2.** Correlations between nasal iris cross-sectional area and pupil/choroidal parameters.

| **N Iris cross-sectional area (µm²) measured 3 mm from the pupil margin and the following parameters** | **SSc group** | | **Control group** | |
| --- | --- | --- | --- | --- |
|  | r | p | r | p |
| **Pupil diameter (µm)** | -0.14 | 0.446 | 0.43 | **0.013** |
| **mTCA (µm²)** | -0.38 | 0.057 | 0.08 | 0.691 |
| **mCVI (%)** | 0.25 | 0.221 | 0.31 | 0.096 |
| **pTCA (µm²)** | -0.1 | 0.603 | 0.04 | 0.835 |
| **pCVI (%)** | -0.18 | 0.341 | 0.07 | 0.719 |
| **pCT Global (µm)** | -0.11 | 0.547 | 0.01 | 0.978 |
| **Choroidal thickness central macular (µm)** | -0.11 | 0.579 | 0.01 | 0.939 |
| **SFCT (µm)** | -0.15 | 0.440 | 0.03 | 0.875 |
| **Choroidal volume central macular (mm³)** | -0.09 | 0.657 | 0.03 | 0.886 |
| **Choroidal volume total (mm³)** | -0.04 | 0.848 | 0.12 | 0.505 |

r – Pearson correlation coefficient

Abbreviations: mCVI – macular choroidal vascularity index, mTCA – macular total choroidal area, pCVI – peripapillary choroidal vascularity index, pCT global – peripapillary choroidal thickness, pTCA – peripapillary total choroidal area, r – Pearson correlation coefficient, SFCT – subfoveal choroidal thickness, SSc – systemic sclerosis, p<0.05 in bold

**2 Intra-group univariate regressions to identify factors associated with the temporal and nasal iris cross-sectional areas measured 3 mm from the pupil margin.**

**Table S3.** Univariate linear regression analysis of factors associated with temporal iris cross-sectional area measured 3 mm from the pupil margin in systemic sclerosis patients.

| **Domain** | **T Iris cross-sectional area (µm²) 3 mm from pupil margin and the following parameters** | **β** | **Standard Error** | **B** | **p** | **R²** | **adj R²** |
| --- | --- | --- | --- | --- | --- | --- | --- |
| **Demographic and clinical parameters** | Age (years) | -1425.000 | 2254.000 | -0.117 | 0.532 | 0.014 | -0.021 |
|  | Sex, male | 31838.000 | 63636.000 | – | 0.621 | 0.009 | -0.027 |
|  | Nicotine, yes | 195611.000 | 86667.000 | – | **0.032** | 0.154 | 0.128 |
|  | MAP (mmHg) | 5160.000 | 2839.000 | 0.324 | 0.081 | 0.117 | 0.081 |
|  | Disease duration (years) | -1572.000 | 3755.000 | -0.078 | 0.679 | 0.006 | -0.029 |
| **Ophthalmological parameters** | AXL (mm) | -8422.000 | 36125.000 | -0.045 | 0.817 | 0.002 | -0.034 |
|  | logMAR | -449390.000 | 435660.000 | -0.188 | 0.311 | 0.037 | 0.002 |
|  | IOP (mmHg) | -1328.000 | 11247.000 | -0.027 | 0.907 | 0.001 | -0.047 |
|  | Iris color (brown/brown-green/green-brown) | -18412.000 | 60527.000 | – | 0.763 | 0.003 | -0.033 |
| **Disease subtype / microvascular involvement (NFC, Cutolo classification)** | dSSc vs lSSc | -45645.000 | 59339.000 | – | 0.448 | 0.021 | -0.014 |
|  | NFC Early vs Active | 44300.000 | 68578.000 | – | 0.524 | 0.037 | -0.034 |
|  | NFC Late vs Active | 66204.000 | 68578.000 | – | 0.343 | – | – |
| **Autoantibody profile** | anti-Scl-70, yes | 63813.000 | 59348.000 | – | 0.293 | 0.044 | 0.006 |
|  | anti-centromere, yes | 15647.000 | 72891.000 | – | 0.832 | 0.002 | -0.038 |
|  | anti-CCP, yes | -249411.000 | 152667.000 | – | 0.115 | 0.096 | 0.060 |
|  | Other Abs, yes | 61961.000 | 59770.000 | – | 0.310 | 0.041 | 0.003 |
| **Organ involvement** | Joint involvement, yes | 14630.000 | 56465.000 | – | 0.797 | 0.002 | -0.033 |
|  | Pulmonary involvement (ILD/fibrosis) | 12018.000 | 61641.000 | – | 0.847 | 0.001 | -0.034 |
|  | Cardiac involvement, yes | 9820.000 | 59934.000 | – | 0.871 | 0.001 | -0.035 |
|  | Digital ulcers, yes | 18281.000 | 59863.000 | – | 0.762 | 0.003 | -0.032 |
| **Inflammation** | CRP (mg/L) | -4017.000 | 13426.000 | -0.057 | 0.767 | 0.004 | -0.038 |
| **Systemic treatment** | PDE5 inhibitors, yes | 27537.000 | 56293.000 | – | 0.629 | 0.008 | -0.027 |
|  | Calcium-channel blockers, yes | 47512.000 | 63286.000 | – | 0.459 | 0.019 | -0.015 |
|  | Hydroxychloroquine, yes | -69196.000 | 69446.000 | – | 0.328 | 0.034 | -0.001 |
|  | Steroids, yes | 43086.000 | 58089.000 | – | 0.464 | 0.019 | -0.016 |
|  | Diuretics, yes | 36011.000 | 61306.000 | – | 0.562 | 0.012 | -0.023 |

*Choroidal and pupil parameters: no significant associations were observed.

β - beta coefficient, B - standardised beta.

Abbreviations: Abs – antibodies, anti-CCP – anti–cyclic citrullinated peptide antibodies, anti-Scl-70 – anti–topoisomerase I antibodies, AXL – axial length, CRP – C-reactive protein, dSSc – diffuse cutaneous systemic sclerosis, HRCT – High-Resolution Computed Tomography, ILD – Interstitial Lung Disease, IOP – intraocular pressure, MAP – mean arterial pressure, NFC – nailfold capillaroscopy, SSc – systemic sclerosis, p<0.05 in bold

**Table S4.** Univariate linear regression analysis of factors associated with nasal iris cross-sectional area measured 3 mm from the pupil margin in systemic sclerosis patients.

| **Domain** | **N Iris cross-sectional area (µm²) 3 mm from pupil margin and the following parameters** | **β** | **Standard Error** | **B** | **p** | **R²** | **adj R²** |
| --- | --- | --- | --- | --- | --- | --- | --- |
| **Demographic and clinical parameters** | Age (years) | -1248.000 | 2248.000 | -0.103 | 0.583 | 0.011 | -0.024 |
|  | Sex, male | 82373.000 | 59448.000 | – | 0.176 | 0.062 | 0.030 |
|  | Nicotine, yes | 234513.000 | 83578.000 | – | **0.009** | 0.214 | 0.186 |
|  | MAP (mmHg) | 2673.000 | 2955.000 | 0.167 | 0.374 | 0.031 | -0.007 |
|  | Disease duration (years) | -3661.000 | 3678.000 | -0.182 | 0.328 | 0.033 | -0.001 |
| **Ophthalmological parameters** | AXL (mm) | -1288.000 | 34465.000 | -0.007 | 0.970 | 0.001 | -0.034 |
|  | logMAR | -323514.000 | 440185.000 | -0.135 | 0.468 | 0.018 | -0.016 |
|  | IOP (mmHg) | -2576.000 | 10299.000 | -0.053 | 0.805 | 0.003 | -0.042 |
|  | Iris color (brown/brown-green/green-brown) | -16859.000 | 59820.000 | – | 0.780 | 0.003 | -0.033 |
| **Disease subtype / microvascular involvement (NFC, Cutolo classification)** | dSSc vs lSSc | -63645.000 | 58421.000 | – | 0.285 | 0.039 | 0.006 |
|  | NFC Early vs Active | 13495.000 | 68205.000 | – | 0.845 | 0.026 | -0.043 |
|  | NFC Late vs Active | 58837.000 | 68205.000 | – | 0.396 | – | – |
| **Autoantibody profile** | anti-Scl-70, yes | 115593.000 | 55343.000 | – | **0.047** | 0.144 | 0.111 |
|  | anti-centromere, yes | 14400.000 | 72635.000 | – | 0.844 | 0.002 | -0.037 |
|  | anti-CCP, yes | -182306.000 | 156697.000 | – | 0.255 | 0.049 | 0.013 |
|  | Other Abs, yes | 75751.000 | 57932.000 | – | 0.202 | 0.062 | 0.026 |
| **Organ involvement** | Joint involvement, yes | 65179.000 | 54425.000 | – | 0.241 | 0.047 | 0.014 |
|  | Pulmonary involvement (ILD/fibrosis) | -9583.000 | 61358.000 | – | 0.877 | 0.001 | -0.034 |
|  | Cardiac involvement, yes | 5701.000 | 59595.000 | – | 0.924 | 0.001 | -0.034 |
|  | Digital ulcers, yes | -6546.000 | 59592.000 | – | 0.913 | 0.001 | -0.034 |
| **Inflammation** | CRP (mg/L) | 14324.000 | 12346.000 | 0.202 | 0.257 | 0.051 | 0.013 |
| **Systemic treatment** | PDE5 inhibitors, yes | -6054.000 | 55744.000 | – | 0.914 | 0.001 | -0.034 |
|  | Calcium-channel blockers, yes | 47799.000 | 63055.000 | – | 0.455 | 0.019 | -0.014 |
|  | Hydroxychloroquine, yes | -62927.000 | 69550.000 | – | 0.373 | 0.027 | -0.006 |
|  | Steroids, yes | 58712.000 | 56155.000 | – | 0.304 | 0.036 | 0.003 |
|  | Diuretics, yes | 50033.000 | 60677.000 | – | 0.416 | 0.023 | -0.011 |

*Choroidal and pupil parameters: no significant associations were observed.

β - beta coefficient, B - standardised beta.

Abbreviations: Abs – antibodies, anti-CCP – anti–cyclic citrullinated peptide antibodies, anti-Scl-70 – anti–topoisomerase I antibodies, AXL – axial length, CRP – C-reactive protein, dSSc – diffuse cutaneous systemic sclerosis, HRCT – High-Resolution Computed Tomography, ILD – Interstitial Lung Disease, IOP – intraocular pressure, MAP – mean arterial pressure, NFC – nailfold capillaroscopy, SSc – systemic sclerosis, p<0.05 in bold

**Table S5.** Univariate linear regression analysis of factors associated with temporal iris cross-sectional area measured 3 mm from the pupil margin in the control group.

| **Domain** | **T Iris cross-sectional area (µm²) 3 mm from pupil margin and the following parameters** | **β** | **Standard Error** | **B** | **p** | **R²** | **adj R²** |
| --- | --- | --- | --- | --- | --- | --- | --- |
| **Demographic and clinical parameters** | Age (years) | -1284.000 | 2797.000 | -0.084 | 0.650 | 0.007 | -0.026 |
|  | Sex, male | 43739.000 | 59583.000 | – | 0.469 | 0.018 | -0.015 |
|  | Nicotine, yes | -9614.000 | 76386.000 | – | 0.901 | 0.001 | -0.033 |
|  | MAP (mmHg) | -46.730 | 5067.060 | -0.002 | 0.993 | 0.001 | -0.045 |
| **Ophthalmological parameters** | AXL (mm) | 41125.000 | 33201.000 | 0.221 | 0.225 | 0.049 | 0.017 |
|  | logMAR | 24041.000 | 1231996.000 | 0.004 | 0.985 | 0.001 | -0.033 |
|  | IOP (mmHg) | 2103.000 | 16262.000 | 0.026 | 0.898 | 0.001 | -0.036 |
|  | Iris color (brown/brown-green/green-brown) | -113859.000 | 65659.000 | – | 0.093 | 0.091 | 0.061 |

*Choroidal and pupil parameters: no significant associations were observed

β - beta coefficient, B - standardised beta.

Abbreviations: AXL – axial length, IOP – intraocular pressure, MAP – mean arterial pressure, SSc – systemic sclerosis, p<0.05 in bold

**Table S6.** Univariate linear regression analysis of factors associated with nasal iris cross-sectional area measured 3 mm from the pupil margin in the control group.

| **Domain** | **N**  **Iris cross-sectional area (µm²) 3 mm from pupil margin and the following parameters** | **β** | **Standard Error** | **B** | **p** | **R²** | **adj R²** |
| --- | --- | --- | --- | --- | --- | --- | --- |
| **Demographic and clinical parameters** | Age (years) | -1917.000 | 2840.000 | -0.122 | 0.505 | 0.015 | -0.018 |
|  | Sex, male | 93055.000 | 58888.000 | – | 0.125 | 0.077 | 0.046 |
|  | Nicotine, yes | -45842.000 | 77447.000 | – | 0.558 | 0.112 | -0.021 |
|  | MAP (mmHg) | -384.200 | 4823.400 | -0.017 | 0.937 | 0.001 | -0.045 |
| **Ophthalmological parameters** | AXL (mm) | 57036.000 | 33105.000 | 0.300 | 0.095 | 0.090 | 0.060 |
|  | logMAR | 632440.000 | 1250747.000 | 0.092 | 0.617 | 0.008 | -0.025 |
|  | IOP (mmHg) | 4308.000 | 16483.000 | 0.052 | 0.796 | 0.003 | -0.034 |
|  | Iris color (brown/brown-green/green-brown) | -107203.000 | 67433.000 | – | 0.122 | 0.078 | 0.047 |

*Choroidal and pupil parameters: no significant associations were observed.

β - beta coefficient, B - standardised beta.

Abbreviations: AXL – axial length, IOP – intraocular pressure, MAP – mean arterial pressure, SSc – systemic sclerosis, p<0.05 in bold
